# Frequency-specific and periodic masking of peripheral characters by delayed foveal input

**DOI:** 10.1038/s41598-024-51710-7

**Published:** 2024-02-26

**Authors:** Nedim Goktepe, Alexander C. Schütz

**Affiliations:** 1 AG Allgemeine und Biologische Psychologie, Philipps-Universität Marburg, Marburg, Germany; 2 Center for Mind, Brain and Behavior, Universities of Marburg, Giessen, and Darmstadt, Marburg, Germany

**Keywords:** Psychology, Human behaviour

## Abstract

The foveal-feedback mechanism supports peripheral object recognition by processing information about peripheral objects in foveal retinotopic visual cortex. When a foveal object is asynchronously presented with a peripheral target, peripheral discrimination performance is affected differently depending on the relationship between the foveal and peripheral objects. However, it is not clear whether the delayed foveal input competes for foveal resources with the information processed by foveal-feedback or masks it. In the current study, we tested these hypotheses by measuring the effect of foveal noise at different spatial frequencies on peripheral discrimination of familiar and novel characters. Our results showed that the impairment of foveal-feedback was strongest for low-spatial frequency noise. A control experiment revealed that for spatially overlapping noise, low-spatial frequencies were more effective than medium-spatial frequencies in the periphery, but vice versa in the fovea. This suggests that the delayed foveal input selectively masks foveal-feedback when it is sufficiently similar to the peripheral information. Additionally, this foveal masking was periodic as evidenced by behavioral oscillations at around 5 Hz. Thus, we conclude that foveal-feedback supports peripheral discrimination of familiar and novel objects by periodically processing peripheral object information.

## Introduction

The visual system is characterized by a division of labor between the foveal and peripheral visual field. Foveal vision allows for high-acuity and color vision in a small part of the visual field and the peripheral visual field allows for a large field of view at lower resolution^1–6^. Interestingly, peripheral and foveal processing are not independent of each other. Peripheral object information is fed back to foveal retinotopic cortex to support peripheral discrimination^7–9^ (for reviews see Stewart et al.^10^; Oletto et al.^11^). An impairment of peripheral object discrimination occurs when foveal-feedback is disrupted by TMS of foveal retinotopic areas^8^ or by asynchronously presenting a foveal stimulus^12^. However, the time window and the precise informational content of this foveal-feedback mechanism is still unclear.

Previous results are somewhat inconclusive about the informational content and the temporal dynamics of foveal-feedback. For instance, peripheral discrimination is impaired when a foveal stimulus, either a noise patch or an object, is presented asynchronously (e.g., Fan et al.^9^). In contrast, when the asynchronously presented foveal object is identical to the peripheral target, it improves peripheral discrimination^12^. However, it is important to note that the paradigm of Yu and Shim^12^ is slightly different than the previous studies, as there was a continuous foveal noise which was briefly replaced by a task critical object. This suggests that the foveal input interferes with the foveal-feedback only when it does not match the peripheral object information processed by the foveal-feedback. This extends previous neuroimaging reports suggesting that the foveal-feedback processing is specific for fine object discrimination. Although, foveal retinotopic cortex is selective to peripheral object information, the similarity between the foveal mask and peripheral objects seems not to be modulating the strength of foveal-feedback impairment^13^. On the other hand, foveal-feedback seems to be processing fine object details and features^9,14^. Yet, it is not clear to what extent the information processed by foveal-feedback is similar to the peripheral object (for reviews see Stewart et al.^10^, Oletto et al.^11^). A plausible explanation accommodating these results would be that the incoming foveal information from the foveal stimulus competes with the foveal-feedback information from the peripheral object for processing resources in foveal retinotopic cortex^13^. This implies that a wide range of foveal stimuli would impair peripheral discrimination and only foveal stimuli identical to the peripheral target would leave peripheral discrimination unaffected or even improved. An alternative explanation is that the foveal information is not competing for foveal resources in general but only selectively masks foveal-feedback information if it is similar to the peripheral object information processed by the foveal-feedback. This explanation takes its inspiration from the principles of spatial frequency processing where visual stimuli mask each other only if they overlap in spatial frequency bands^15–20^. Therefore, according to the masking explanation, the efficacy of a foveal stimulus in impairing foveal-feedback depends on the similarity between the peripheral and the foveal information. However, when the foveal stimulus is identical to the peripheral object information processed by foveal-feedback, the foveal stimulus no longer acts as a mask and aids foveal-feedback as a secondary source of object information. Current results do not distinguish if there is a more general competition or a selective masking between the foveal stimulus and the foveal-feedback.

Characters of written language present a unique opportunity to test these explanations and identify the informational content of foveal-feedback. Characters are one of the best studied visual objects and are highly relevant for daily life^20–24^. On the one hand, they are more complex than simple visual objects such as oriented gratings or moving dots, which are immune to foveal-feedback impairments^9,12^. On the other hand, they are still simple objects in the sense that they are identified in a common narrow spatial frequency band with a mean of 3 cycles/letter ^20,21^. This makes them particularly useful to understand if the foveal object is competing with or masking the foveal-feedback information. If the information processed by foveal-feedback is masked by the foveal object, then a foveal object that contains similar spatial frequencies as the peripheral object would impair the peripheral discrimination more than a foveal object that overlaps less in spatial frequencies. In contrast, if the foveal object is competing with the foveal-feedback information, then foveal objects would impair peripheral discrimination in a similar manner regardless of the overlap in spatial frequency bands. Thus, we tested the predictions of the “masking” and “competition” explanations by using foveal noises defined by different frequency bands.

The estimated time windows of foveal-feedback vary considerably across previous studies (for a comparison see Table 1 in Oletto et al.^11^), potentially due to having low temporal resolution. To achieve a higher temporal resolution in the current study, we densely sampled the stimulus onset asynchrony (SOA) of the foveal noise. Another reason that might have contributed to this variation might be the different analytic approaches used to identify the time window of foveal-feedback. One group of studies used performance in peripheral discrimination without a foveal mask/stimulus as a benchmark for the foveal mask trials with different SOAs^9,14,25^. This approach has the advantage of directly testing the effect of a foveal mask on peripheral discrimination. However, it rests on the implicit assumption that any performance difference between the foveal mask trials and the no mask trials are due to foveal-feedback. Therefore, it is prone to false positives if other factors affect performance. The second group of studies compared the different foveal mask SOA conditions relative to each other to identify the effective time window of foveal-feedback. This approach rests on the implicit assumption that foveal feedback is a transient effect, occurring only at some SOAs^13,26^. Therefore, it is prone to false negatives, especially if the foveal-feedback impairment spans across multiple SOAs. In Experiment 1, we followed the latter approach and compared the change in discrimination performance across SOAs using bootstrapping. Finally, a recent study has shown that the variation in the estimated time windows might be also due to foveal-feedback-feedback not having one but multiple critical time windows of reduced peripheral discrimination oscillating at 3 and 12 Hz^25^. Another reason to densely sample SOAs is the fact that the speed of visual processing depends on the spatial frequencies of the signal. This is captured by coarse-to-fine visual processing where low spatial frequency information is processed before high spatial frequency information (for a review see Kauffmann et al. ^27^). Therefore, depending on the frequency composition of the peripheral object, the time window of foveal-feedback might shift. Similarly, the frequency composition of the foveal stimuli could also determine when the foveal information arrives at the foveal retinotopic cortex and intercepts with the foveal-feedback processing of the peripheral object. Thus, the time window of the impairment in peripheral discrimination might vary depending on when the foveal and foveal-feedback information meet in foveal retinotopic cortex.

Finally, foveal-feedback has been studied almost exclusively with practically novel, artificial objects^13,26^ or familiar, everyday objects^14^. Although foveal-feedback was present for both types of stimuli, none of the previous studies compared them directly in the same experimental design. Such a comparison is complicated by the large differences in low-level properties of the employed stimuli. Here, we directly compare an entirely novel set of characters (Korean) to over-trained characters (Latin) to check if foveal-feedback applies to both classes of characters. Transsaccadic feature prediction is another mechanism that supports peripheral object discrimination by using both peripheral and foveal information. It supports peripheral discrimination by generating object predictions using previously associated peripheral and foveal object information^28–30^. We recently showed that peripheral discrimination of novel objects similarly benefits from these predictions albeit in a more general way and to a weaker extent^31^. To check if foveal-feedback applies to novel and familiar objects similarly, we compared Korean characters that were novel to our observers and Latin characters that were over-trained through lifespan.

## Results

### The temporal dynamics and the informational content of foveal-feedback

**Figure 1:**
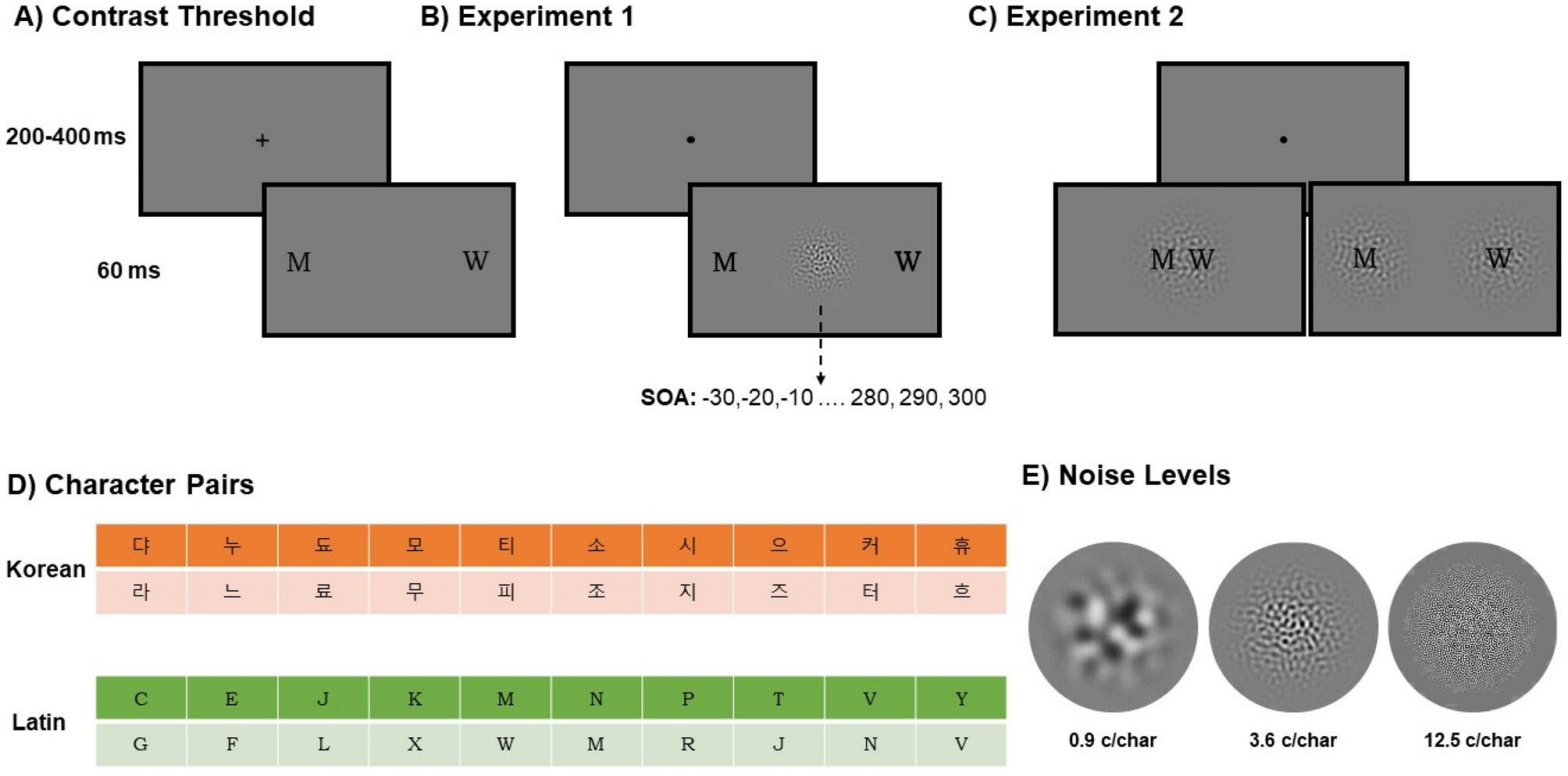
Stimuli and Methods. Example of trials from contrast threshold (**A**), Experiment 1 (**B**), and Experiment 2 (**C**). A, Experiment 1 used two QUEST procedures to determine the character contrast threshold of each observer (see “Methods”). (**B**) In Experiment 1 observers fixated to a central dot for a random duration of 200–400 ms. Following the offset of the fixation dot, either two Korean or Latin characters were presented 10° left and right of the fixation location for 58.33 ms. Low, Medium, or High level noise was presented at fixation for 58.33 ms at a stimulus onset asynchrony (SOA) of -30 to 300 ms. (**A**–**C**) Stimuli are not drawn to scale for illustration purposes. (**D**) List of Latin and Korean character pairs that constituted different pairs in the respective conditions. The entire set of character used in the experiment can be found in “Methods”. (**E**) Three frequencies of noise that were used in the experiments. As the width of the characters was approximately one degree of visual angle, one c/char_width_ corresponds to one c/deg.

The main purpose of the current experiment was to investigate the information that is processed by foveal-feedback, how it interacts with foveal input (e.g., masking vs. competition) and to check if foveal-feedback generalizes to familiar peripheral targets. To this end, we asked observers to perform a peripheral same-different judgment task using characters from their native script (Latin alphabet) and a foreign script (Korean). A bandpass-filtered foveal noise centered on one of three frequency bands was presented at different SOAs (see Fig. 1). Thus, any difference across foveal noise types provides evidence for masking of foveal feedback. Similarly, any difference between Latin and Korean characters would provide evidence for a specific role of foveal-feedback for novel or familiar objects.

For each character and noise condition, we densely sampled a relatively wide time range of SOAs to determine the time window of foveal-feedback processing with a resolution much higher than most of the previous studies. For this, we measured discrimination of peripheral characters with asynchronous foveal noises at SOAs separated only by 8.33 ms. However, due to the presentation duration of 58.33 ms, the foveal noise in neighboring SOAs overlapped in time, similar to a recent study^25^ that also followed a dense sampling procedure between 0–500 SOA. One key difference in our approach is that we calculated discrimination performance (d’) for each SOA, and then aggregated all SOAs where the foveal noise was present during a given time window (for details see “Data Analysis”). Thus, our approach of aggregating overlapping foveal noise presentation estimates the effect of foveal noise presented at different time points relative to the onset of the peripheral stimuli (*time relative to peripheral onset* hereafter, TPO). In other words, our analysis shows at which time interval foveal noise impairs peripheral discrimination of characters. It is important to note that this approach of analyzing overlapping foveal noises together rests on the assumption that the effect of foveal noise on foveal-feedback is not limited to its onset but sustained for a longer period throughout its presentation, as it has been observed for masking^32,33^.

**Figure 2:**
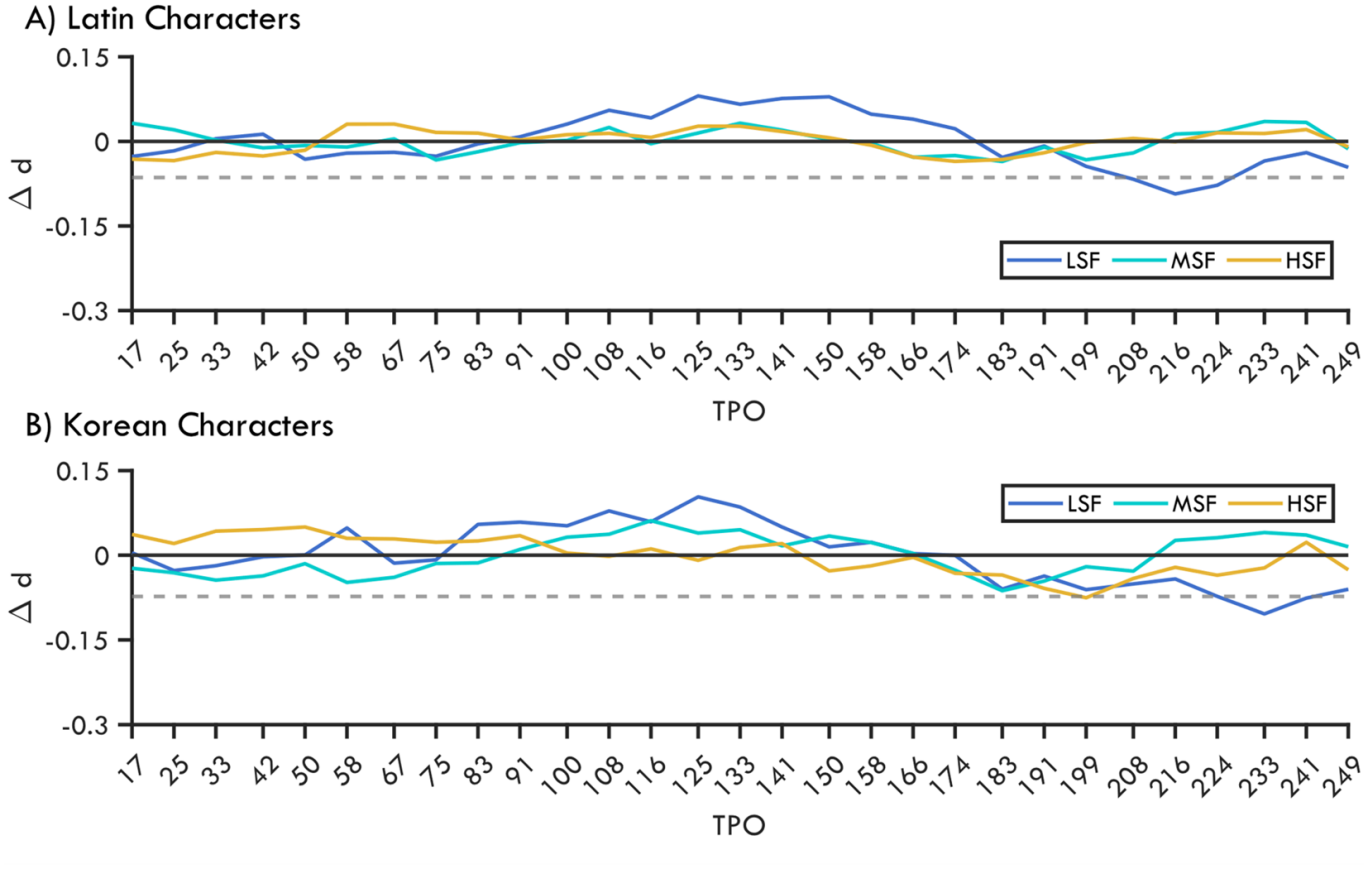
Change in peripheral discrimination performance (*d′*) for Latin (**A**) and Korean (**B**) characters with low-, medium-, and high-spatial frequency foveal noise as a function of time relative to peripheral stimuli onset (TPO). Negative *d′* values indicate discrimination performance below the mean. The dashed line shows the 95th percentile of the permuted performance corresponding to one-sided statistical test (for illustration purposes only the smallest critical value is shown). The lower horizontal bars indicate SOAs with significant reductions in performance.

Following previous studies^13,26^, we identified the time window of foveal-feedback by checking the change in the relative discrimination performance across noise intervals within each noise and character conditions. To this end, we created 10,000 permuted-datasets by shuffling the SOA labels of each trial separately for each observer and conditions. For each empirical and permuted-dataset in each noise and character condition, we set the mean discrimination performance of each participant to zero by subtracting their mean discrimination performance from their performance for each SOAs under the same condition. Hence, negative values in Fig. 2 indicate a reduction in performance compared to the mean. The top 95th percentile performance of the permuted-datasets in each condition (dashed lines in Fig. 2) served as the critical value of the underlying omnibus statistical test that is equivalent to a one-sided test at α = 0.05.

The temporal profile of peripheral discrimination was remarkably similar for each character and noise condition. In both conditions, discrimination performance in all noise conditions was lower than the no-noise baseline condition (see Supplementary Materials S1). The peripheral discrimination performance appeared particularly vulnerable to foveal noise for the period of 90 ms after the stimulus onset (0 TPO). During the first 58.33 ms of this period, the foveal stimulus and peripheral targets were both present at the same time. Therefore, this period of vulnerability could be also attributed to divided attention^9^. The following period between 90–170 was characterized by a relatively stronger peripheral discrimination performance. Following the period of recovery, the discrimination performance declined again at 180 ms after stimulus onset. The interval between 180–190 ms was a period of major reduction in all conditions, which overlaps with the effective time windows reported by multiple studies (Yu and Shim^12^, 150–200 ms; Fan et al.^9^, 150–233 ms; Weldon et al.^13^ 117–234 ms; Weldon et al.^26^ 117–234; Contemori et al.^25^ 94, 157 ms). However, the period of significant reduction we observed was slightly later around 220 ms and was present almost exclusively for the LSF noise condition. Peripheral discrimination of Latin characters was significantly impaired by LSF noise at 208 ms (*p* = 0.043), 216 ms (*p* = 0.01), and 224 ms (*p* = 0.041). Similarly, discrimination of Korean characters was significantly impaired at 233 ms (*p* = 0.01) and 241 ms (*p* = 0.045) by LSF noise. In addition, a HSF foveal noise at 199 SOA significantly impaired the discrimination performance of Korean characters (*p* = 0.045). At a first glance, this significant dip after the common 180–190 interval looks at odds with previous results. However, previously reported foveal-feedback time windows were mostly from studies with lower temporal resolution, which used long foveal presentations and few SOAs. Thus, our noise sampling allowed us to detect critical periods within the reported periods with higher precision. Hence, like the major dip at 180 ms, the significant reduction period around 220 ms is also consistent with previous reports and maybe the epicenter of the foveal-feedback period. Critically, while the reduction of 180 ms was common in all noise conditions, the significant reduction around 220 ms was specific to LSF noise.

We then compared the strength of foveal noise modulation across noise conditions separately for each character condition. To this end, we used the TPOs that yielded the strongest impairment in each noise condition. This allowed us to avoid any temporal bias in the analysis as different spatial frequencies could mask foveal-feedback at different time intervals. By means of shuffling noise condition labels for each participant we obtained 10,000 permuted-datasets (for details see “Data Analysis”). Next, we checked if the differences between the observed conditions were more extreme than the central 95th percentile of differences in the respective differences in the permuted-datasets. Thus, for each noise comparison we tested significance with a two-tailed statistical test at α = 0.05. We found that for Latin characters, LSF noise impaired performance significantly stronger than MSF (*p* = 0.047) and HSF noise (*p* = 0.007). There was no significant difference between MSF and HSF noise (*p* = 0.51). The discrimination performance for the Korean characters followed the same trend but did not reach significance.

So far, our analysis compared differences in discrimination performance between TPOs. Comparing discrimination performance in trials with different TPOs essentially checks if there are temporally limited periods of major reductions. This is highly desirable as the foveal noise could impact discrimination performance in other ways, for instance as a distractor^9^. However, it is a conservative method that would completely miss an effect of periodic reductions in multiple time intervals. To capture potential periodic reductions, we conducted a complementary frequency analysis reported in the next section.

### Periodicity of foveal-feedback

**Figure 3:**
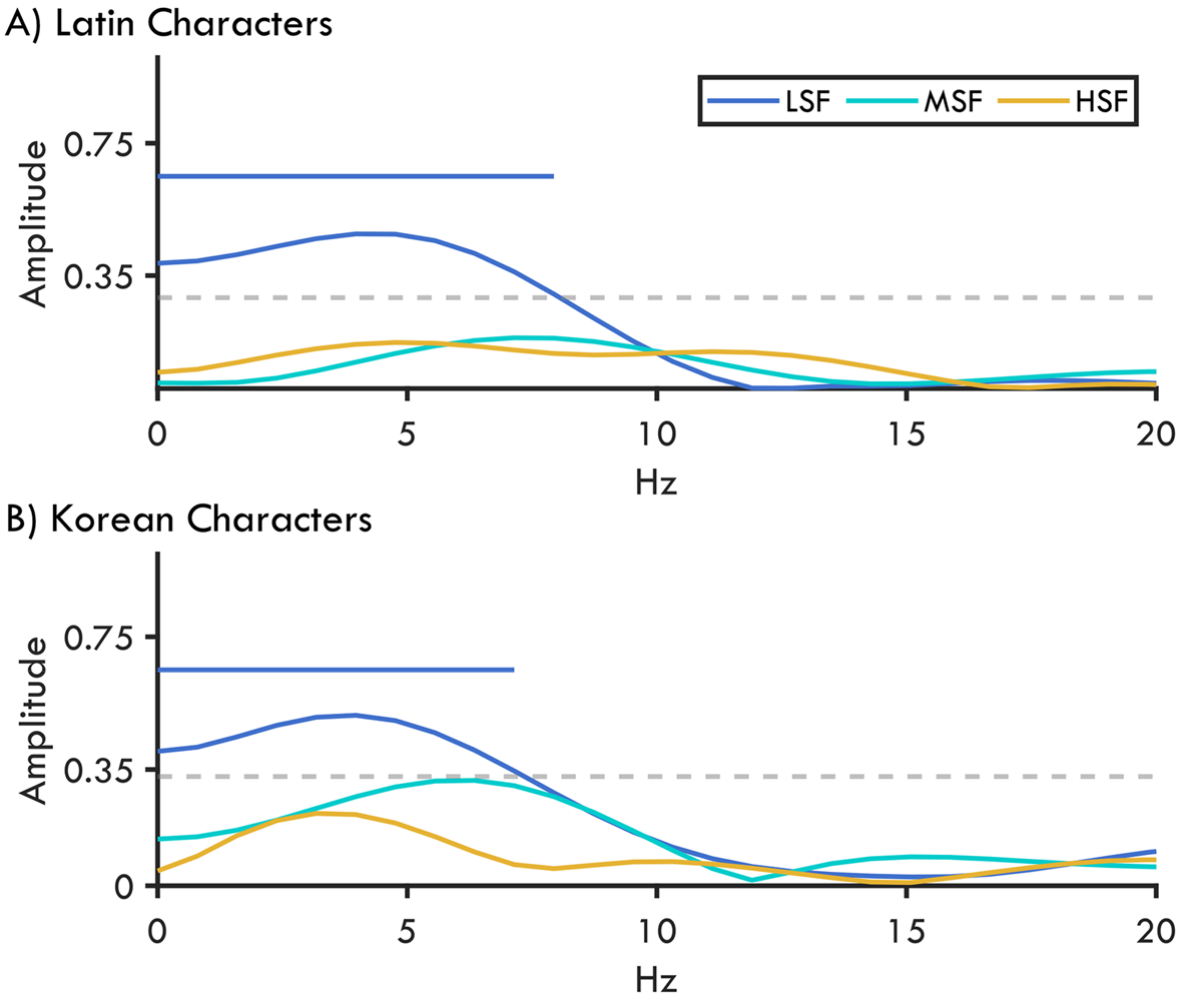
Perceptual performance oscillations for Latin (**A**) and Korean (**B**) characters across LSF, MSF, and HSF conditions. Dashed line shows 95th percentile amplitude calculated from permuted data corresponding to a one-sided statistical test (for illustration purposes only the largest critical value is shown). Horizontal bars indicate frequencies with a significantly larger amplitude.

It has been recently suggested that foveal-feedback is a recurrent process instead of a one-off occurrence^25^. The periodicity of a mechanism is typically measured by analyzing the oscillatory signals from EEG or from perceptual performance^34–39^. Like neural oscillations, behavioral oscillations are analyzed using Fourier transformations to estimate the contribution of each frequency band. Contemori et al.^25^ showed that the influence of a central noise on the peripheral discrimination performance oscillates at around 3 and 12 Hz. In the current study while we employed a similar sampling approach, we collapsed the overlapping SOAs together, such that the effect of foveal noise overlapping in time are considered together within a smaller temporal range. We found that LSF noise is more effective than MSF and HSF noise. Therefore, if the behavioral oscillations reported earlier underly foveal-feedback processes, then we should find similar oscillations to be stronger in the LSF condition. To link foveal-feedback to the periodic changes in the perceptual performance, we analyzed the Fourier spectrum of each character and noise condition. In order to identify significant peaks in amplitudes, we generated permuted amplitudes from the shuffled-datasets and performed a one-tailed omnibus statistical test with α = 0.05 for each noise condition (see “Data Analysis” for details). We found frequency amplitudes peaking at around 5 Hz for LSF and MSF noise for both character conditions. As expected, in the LSF condition, amplitudes for these frequencies were significantly higher than the permuted amplitudes (dashed lines in Fig. 3). For the Latin characters these frequencies ranged from 0.79 to 7.94 Hz (*p*_*max*_ = 0.045, *p*_*min*_ = 0.006). For Korean characters we found that similar frequencies from 0.79 to 7.14 Hz (*p*_*max*_ = 0.044, *p*_*min*_ = 0.009) had significantly higher amplitudes.

Next, we compared the amplitudes across noise conditions by analyzing the Fourier spectrum of the permuted data generated for the noise comparisons (for more details see “Data Analysis”). Then, we calculated the amplitude differences between the maximum amplitude in each real and permuted noise condition. Next, we used the central 95th percentile of the permuted differences to assess statistical significance at α = 0.05 with a two-tailed test. We found that for the discrimination of Latin characters, the LSF noise condition had stronger oscillations than MSF (*p* = 0.007) and HSF (*p* < 0.001). The amplitude difference between MSF and HSF noise conditions did not reach significance (*p* = 0.424). For Korean characters, LSF noise had the highest amplitude, but it was only significantly stronger than HSF (*p* = 0.005).

Overall, we found a significant reduction of discrimination performance around the TPO of 220 ms for the LSF noise condition for both character conditions. Therefore, we suggest that foveal-feedback is also supporting the peripheral discrimination of novel objects. LSF noise was particularly detrimental to discrimination performance and led to stronger impairments. This frequency-specific effect is more compatible with masking rather than competition or attention^9^.

### Foveal-feedback follows spatial frequency processing

Previous studies provided some evidence that the informational content of foveal-feedback is specific at least at the level of object category^7,9,12^ (but see Weldon et al.^13^). Moreover, foveal-feedback facilitates the processing of finer features of the peripheral objects such as orientation^9^ or subordinate category differences^14^ (for reviews see Stewart et al.^10^; Oletto et al.^11^). So far, our results suggest that peripheral discrimination performance of characters was particularly impaired by LSF noise acting as a mask. The specific effect also implies that foveal-feedback processes information that is more vulnerable to a specific type of foveal input. This systematic impairment due to masking of LSF noise provides us with a unique opportunity to better describe what is exactly processed by foveal-feedback. The superiority of LSF noise over MSF and HSF can be explained by fundamental spatial frequency processes^20–24^. Within this framework, the most effective foveal noise should be the most effective mask for the information processed by foveal-feedback. This means that foveal-feedback is processing object information that has a spatial frequency distribution very similar to LSF noise. Considering previous evidence suggesting that the foveal-feedback is processing peripheral object information, LSF noise should be masking Korean and Latin characters more efficiently than the MSF or HSF noise. If LSF noise is better at masking foveal-feedback information, then our peripheral characters should be processed by the same frequency channels that are responsible for processing LSF noise. Hence, if it is the precise object information encoded by peripheral retinae processed by foveal-feedback, then LSF noise should be better at masking peripheral characters than other frequency bands.

It is well established that character processing in the fovea occurs at a higher spatial frequency band, equivalent to our MSF noise^20^. Therefore, if the peripheral characters are masked by the foveal noise according to foveal processing of letters, the strongest performance reduction would be expected for MSF instead of LSF noise. However, peripheral processing of letters is supported by frequency channels closer to LSF than MSF noise^40,41^. Hence, if the foveal noise is masking the peripheral information processed by foveal-feedback, then LSF should be a more effective mask because the spatial frequency composition of the peripheral information is more similar to LSF than MSF. Thus, we conducted a follow-up experiment to test whether peripheral LSF or MSF noise is a better mask for peripheral character discrimination. We measured the discrimination performance at 0° (fixation) and 10° eccentricity for Latin and Korean characters (Fig. 1). Both characters were embedded in LSF or MSF noise with the same RMS contrast. Then, using a staircase method, we varied the character contrast to determine the discrimination threshold of observers.

**Figure 4:**
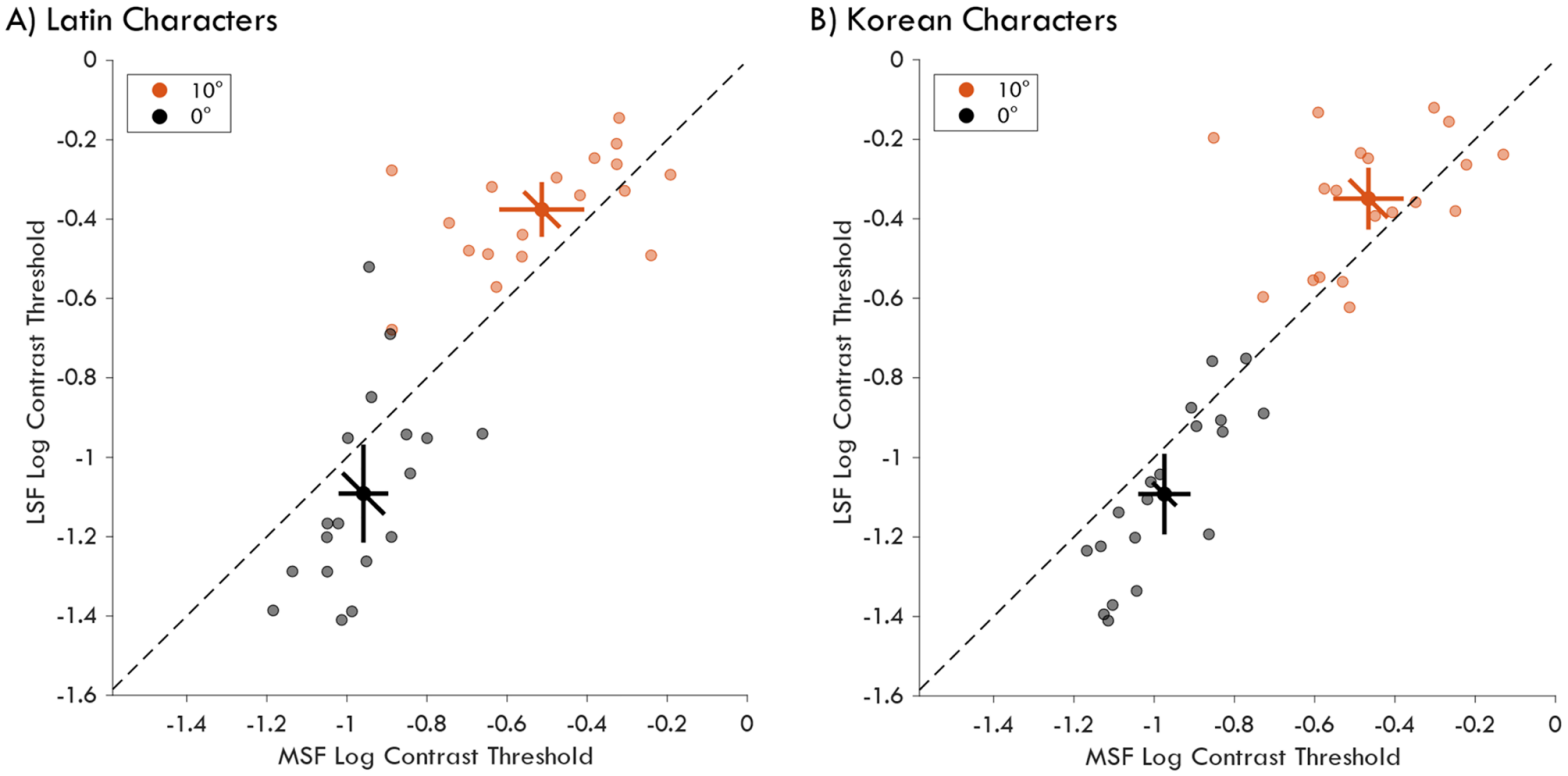
Character contrast thresholds for Latin (**A**) and Korean (**B**) characters at 0° (fixation) and 10° eccentricity. Light colors indicate individual participants and dark color the average across participants. Error bars represent 95% confidence intervals for MSF noise (horizontal), LSF noise (vertical), and their pairwise comparisons (diagonal).

Figure 4 shows character contrast thresholds of each observer for peripheral and foveal discrimination judgments of characters embedded in LSF or MSF noise. For both character conditions, character contrast thresholds were higher for LSF noise than MSF noise for targets presented at 10° eccentricity. To compare contrast thresholds against both noise and character conditions we used a Linear Mixed Model (LMM), where character, noise frequency, and stimulus eccentricity were fixed factors and participants were random factors. The model yielded significant main effects of stimulus eccentricity (*t*(119) =  − 13.6, *p* < 0.001) and noise frequency (*t*(119) =  − 2.136, *p* = 0.035) but no effect of character (*t*(199) =  − 0.394, *p* = 0.694). Crucially, there was a significant interaction between noise frequency and target eccentricity suggesting that the effectiveness of noise masking depended on stimulus eccentricity (*t*(119) = 3.031, *p* = 0.003, see “Methods” for complete statistical results). Post-hoc Holm-Bonferroni corrected comparisons on eccentricity and noise interaction showed that contrast thresholds were higher for MSF than LSF noise at fixation (*p* = 0.011), as expected. Crucially, in line with previous studies^40,41^ the pattern was reversed for characters presented at 10° eccentricity and contrast thresholds were higher for LSF than for MSF noise (*p* = 0.009). Hence, we suggest that the effectiveness of foveal noise at impairing peripheral discrimination depends on the efficacy of the noise for peripheral processing and not for foveal processing. Moreover, our results provide the first direct evidence that foveal-feedback is processing peripheral information that is vulnerable to masking.

## Discussion

The foveal-feedback mechanism supports peripheral object discrimination by processing information related to peripheral objects in foveal retinotopic visual cortex^7,9^. Previous studies provided mixed evidence about the informational content of foveal-feedback and its relation to peripheral objects. It was not clear whether the input from the foveal object competes or masks the information processing by the foveal-feedback. In the current study, we tested these hypotheses by varying the relationship between the peripheral target and the foveal noise. According to the competition hypothesis, all foveal noises should have comparable effects on peripheral discrimination as they all compete with foveal-feedback. In contrast, according to the masking hypothesis, the similarity of the foveal and peripheral objects should dictate how foveal-feedback will be affected. Our results favor the latter argument as the foveal-feedback impairment was stronger for noise with low- than with medium- or high-spatial frequency. In Experiment 2, we showed that the foveal-feedback follows spatial frequency processing of peripheral vision as low-spatial frequency noise was more efficient at masking peripheral information than medium-spatial frequency noise.

Previous studies described foveal-feedback as a mechanism supporting the discrimination of complex objects^9,12^, but not simple visual objects. While higher-level objects such as rendered complex objects^13,26^ or complex objects from daily life^14^ are clearly affected by masking of foveal-feedback, the discrimination of simple visual features like motion^9^ and orientation^12^ does not seem to be masking of foveal-feedback in the same way. Our results suggest that the masking of foveal-feedback processing not only depends on the properties of the peripheral information but also on the relationship between peripheral and foveal information. Thus, we argue that foveal-feedback may not be selective in which type of object it supports, instead it might be processing all kinds of objects but with varying degree. The degree of support might depend on factors such as how resolvable the peripheral object is by peripheral vision alone and the spatial frequency composition. The absence of foveal-feedback for simple features could also be caused by using noise that was not effectively masking the foveal-feedback processing for those features. The involvement of foveal-feedback in general object processing is compatible with the suggested “visual sketchpad” functionality of foveal-feedback^8,13,25^ (for a review see Oletto et al.^11^). Within this framework, foveal inputs that are more similar to the visual sketchpad would intrude foveal-feedback more readily than less similar inputs.

Another topic of debate is the time window of foveal-feedback. The majority of previous studies presented foveal noise for long presentation durations at few, temporally spread-out SOAs, allowing only for a coarse temporal estimation of foveal-feedback. In Experiment 1 we used overlapping and dense sampling of SOAs to obtain a more precise estimation. Similar to a recent study by Contemori et al.^25^, we used overlapping and dense sampling of SOAs in Experiment 1 to obtain a more precise estimation of the time window of foveal-feedback. Unlike Contemori et al.^25^, we analyzed SOAs with overlapping foveal noise presentations together to estimate the effect of foveal noise at different TPOs. With this finer sampling method, we showed that previous seemingly inconsistent reports of foveal-feedback onsets might be in fact consistent with one and another.

Our results suggest that the LSF noise impairs the discrimination of Korean characters at a later time point than Latin characters. Considering that our observers were not experienced with Korean characters, the difference in foveal-feedback timing might be reflecting the difference in task difficulty^9^. Alternatively, the timing difference between Korean and Latin characters could be caused by different origins of the foveal-feedback. Two previous neuroimaging studies found parallel object selectivity in foveal retinotopic and lateral occipital complex (LOC) and argued that feedback might be originating from LOC^7,9^. However, both studies used complex objects as stimuli, which makes LOC a good candidate for feedback. It is possible that higher-order feedback signals can originate from different areas depending on the kind of information that is processed. For instance, numbers and letters are represented by partially distinct neural pathways despite their high visual similarity^42–45^. Hence, foveal-feedback of Latin characters might originate from letter specific areas rather than LOC. On the other hand, given the inexperience of our observers, Korean characters might be treated as complex objects rather than letters, thus fed back from LOC. Neuroimaging studies could investigate the origin of these feedback signals and distinguish foveal-feedback from other mechanism that seemingly offer same functionality.

More fundamental questions for future research are what foveal retinotopic cortex brings to the table by reprocessing peripheral information that is restricted to the resolution of the peripheral visual field. For instance, prior foveal processing of objects facilitates their peripheral processing and reduces crowding^46^. Another potential role of foveal-feedback is to provide a memory buffer or a sketchpad for storing fine detailed object information for re-evaluation^13^ (see Oletto et al.^11^ for a comprehensive review). A recent study^25^ showed that observers become more conservative following foveal-feedback impairment, which implies some top-down control on peripheral discrimination. Considering the interaction between foveal and peripheral visual fields, one key question is how the visual system bridges processing differences such that information can be exchanged efficiently. Previous studies^28–31^ showed that the visual system can learn object-specific associations that connect peripheral and foveal object information. This mechanism supposedly helps to align the peripheral and foveal representation of objects and to compensate for the differences in processing. This object-specific mechanism is supplemented by a more generative transformation between peripheral and foveal information because peripheral-to-foveal matching is also possible for completely unfamiliar objects^31^. Our finding of foveal-feedback effect for both, highly-overtrained (Latin characters) and novel (Korean characters) objects suggests that these peripheral-foveal associations and foveal feedback processing are somewhat independent of each other. On the one hand, foveal-feedback processing supports peripheral discrimination even when there is extensive experience and as a consequence, strong peripheral-foveal associations for a certain stimulus like the Latin characters in our study. This means that the presence of strong peripheral-foveal associations does not render foveal-feedback processing superfluous. On the other hand, foveal-feedback processing can support peripheral discrimination even when the objects are novel and have not been observed in dynamic, transsaccadic conditions like the Korean characters in our study. This means that peripheral-foveal associations are not necessary to accomplish foveal-feedback processing.

In conclusion, we found that foveal noise is acting like a spatial frequency mask for the foveal-feedback processing of peripheral objects. This means that the processing of foveal-feedback depends on the spatial-frequency composition of the peripheral information rather than on the spatial-frequency preference of foveal processing. The disruption of foveal-feedback was detrimental for novel and familiar objects alike, which means on the one hand that foveal-feedback processing does not require specific peripheral-foveal associations and on the other hand that experience with an object does not render foveal-feedback processing superfluous. In sum, we describe foveal-feedback as a mechanism that supports peripheral discrimination of both familiar and novel objects by periodically processing peripheral object information.

## Methods

### Participants

A total of 36 observers including the author N.G. completed Experiment 1. 26 observers (19 females, mean age = 24.38 ± 6.02) performed the *Korean characters* condition and 31 observers (22 females, mean age = 25.26 ± 5.65) performed the *Latin characters* condition.

In Experiment 2, 26 observers (17 females, mean Age = 27.38 ± 5.28) performed the discrimination task with both Korean and Latin characters. After exclusions, the analysis included the data of 19 observers (13 females, mean Age = 26.74 ± 5.62) for Korean and 18 observers (11 females, mean Age = 27.22 ± 5.7) for Latin characters. Korean and Latin character conditions were measured independently in different groups of observers. Coincidently, 21 observers performed both conditions. The order of measurement for these observers was not randomized as the effect of different foveal noises was assessed separately for the two character conditions. In total 9 out of 21 observers completed the Korean characters condition before the Latin characters condition.

All observers had normal or corrected to normal vision without any known visual condition. They all signed informed consents prior to testing and received monetary compensation. The experiment was approved by the local ethics commission of the Department of Psychology of Marburg University (proposal number 2015-35k) and were conducted in accordance with the Declaration of Helsinki (1964).

### Stimuli

A subset of modern Latin characters (A, C, D, E, F, G, H, J, K, L, M, N, P, R, T, U, V, W, X, Y) and a subset of Korean characters (댜, 누, 됴, 모, 티, 소, 시, 으, 커, 휴, 버, 쿄, 라, 느, 료, 무, 피, 조, 지, 즈, 터, 흐, 비, 크) rendered in the Bookman Old Style font served as stimuli. Each character subtended an area of approximately 1 × 1° on the display. For “same” trials in one block, 10 characters were randomly drawn from the respective character pool. For “different” trials, a set of 10 character pairs was used for all observers (see Fig. 1 for the pairs). Low (LSF), medium (MSF), and high (HSF) spatial frequency noises subtended an area of approximately 6.5° × 6.5° and were bandpass (Gaussian) filtered random pink noises. Filtered noises had central frequencies of 0.9, 3.6, and 12.5 c/character and a band width of 1.6 octaves. Therefore, there was no frequency overlap between the three types of noise. They were rendered at fixation within a Gaussian envelope with unit standard deviation. They were displayed at a 20% root mean square contrast (RMS), which means that the noise luminance had the same mean luminance as the background but varied by 20% standard deviation. The RMS contrast is more suitable for comparing stimuli with different spatial frequencies than Michelson contrast^47^. In Experiment 2, the same characters and noises were used with the following differences: The characters were displayed at various contrasts to measure contrast thresholds. Noises were used as a background for foveally or peripherally presented characters at 5% RMS contrast.

### Equipment

The stimuli were displayed at a 106 cm distance on a back-projection system comprising of a 120 Hz PROPixx projector (VPixx Technologies, Saint Bruno, QC, Canada) with 1920 × 1080 resolution and a 91 × 51 cm Stewart Filmscreen (Torrance, CA). Prior to the experiment, gamma and hot spot corrections were carried out to ensure linear luminance output. After calibration, the display had a luminance of 2.07, 71, and 140 cd/m^2^ for black, grey, and white pixels, respectively. An Eyelink 1000+ (SR Research Ltd., ON, Canada) was used to record eye movements. Eye movements were recorded at 1000 Hz sampling rate after a 9-point calibration. Calibration points were positioned at the screen center and 5 degrees around it. The experiment was programmed in MATLAB (MatWorks. Inc.) using Psychtoolbox^48,49^ and EyelinkToolbox^50^. In all experiments, participant responses were collected via keyboard. Participants used the left and right arrow keys to indicate that the characters in a pair were the same or different, respectively.

### Procedure

#### Experiment 1

In Experiment 1, both Korean and Latin characters conditions followed the same procedure and were each completed in three separate sessions. Each session consisted of a thresholding block and an experimental block.

The thresholding procedure contained two QUESTs^51^ to determine individual character contrast values necessary for achieving 75% accuracy without a foveal noise (see Supplementary Fig. S2A). At the beginning of each trial, a fixation cross was presented for a random duration of 200–400 ms on a neutral background. Following the fixation offset, two characters were presented at 10° on the horizontal meridian. Observers were asked to indicate whether these characters were the same or different. Observers were instructed to keep fixation at the center for the full duration of the trial. The mean of the two contrast values suggested by QUESTs was used in the experimental block. Following the eye tracker calibration, observers proceeded to the experimental trials.

Experimental trials consisted of no-noise (baseline), LSF, MSF, and HSF conditions. The no-noise condition was similar to the thresholding trials, but with a fixed stimulus contrast. In the noise trials, a foveal noise was presented at a random stimulus onset asynchrony (SOA). At the beginning of each session SOAs between − 25 and 300 ms were randomly selected from a uniform distribution and repeated equally in each noise condition. An SOA of zero reflects simultaneous onset of the stimulus and noise. Hence, for negative SOAs, the noise preceded the stimulus. Each trial started with a fixation check followed by a fixation cross presented for a random duration between 200–400 ms. 58.33 ms after the offset of the fixation cross, the characters were presented for 58.33 ms. For noise trials, the foveal noise was presented at the respective SOA for 58.33 ms. If the gaze of observers left a central 6.5 × 6.5° square at any time during the trial, they received an auditory warning at the end of that trial. Each session consisted of 80 threshold trials and 620 test trials (i.e., 20 no-noise trials, and 200 trials per noise condition) in randomized order, which took about two hours.

#### Experiment 2

Experiment 2 used a 2-up-1-down staircase procedure to determine the discrimination threshold for characters directly embedded in either LSF or MSF noise presented at 0° (fixation) or 10°. The embedded peripheral characters were at the center of a 6.5° × 6.5° LSF or MSF noises, whereas centrally presented characters were approximately 0.7° degrees apart and embedded within LSF or MSF noise (6.5° × 6.5°). After giving their consent, Experiment 2 started with eye tracking calibration. Characters from each condition were presented in blocks. Each block was further divided into two subblocks for the foveal and 10° eccentricity conditions. Each block contained one ascending and one descending staircase. The staircases were interleaved and the character contrast was reduced after two consecutive correct responses or increased after an incorrect response. The order of blocks and subblocks was randomized. Each trial started with a fixation check, followed by the 58.33 ms presentation of a pair of characters within one of the two noise backgrounds. Observers again performed a same-different judgment. The experiment had 640 trials and took about 1.5 h.

### Data analysis

#### Experiment 1

Trials with erroneous fixations were discarded (< 1% of trials per observer). In Experiment 1, for each character and foveal noise condition, noise trials were assigned to SOAs based on the vertical retrace time stamps of the display. The refresh rate of the display (120 Hz) did not allow to sample a comparable number of trials for the − 10 SOA. Therefore, these trials were also discarded (< 2% of all trials). Then, we calculated the sensitivity index (d’) for each SOA in each foveal noise condition. The sensitivity index (d’) was calculated using Hautus correction^52^ with the following formula: d’ = z((hits + 0.5)/(hits + misses + 1)) − z((false alarms + 0.5)/(false alarms + correct rejections + 1). We ran a moving average with a 58.33 ms (7 frames) window to pool together temporally overlapping foveal noise presentations to calculate the impact of foveal noise at the time relative to peripheral stimuli onset (TPO). Thus, for calculating the bin at 50 ms SOA, trials with noise onset asynchronies from 50 to 108.33 ms were used.

For TPO comparison, we used an omnibus one-tailed permutation test. For this, we created 10,000 permuted datasets by reshuffling the perceptual responses across trials of the same condition and observer. Permuted datasets were then analyzed in the same way as the actual dataset. For each SOA in each noise and character condition, the top 95th percentile of the permuted datasets was used as the critical value. Any SOAs with an observed value less than the critical value were deemed significant at α = 0.05 for a one-tailed test.

For comparing the strength of impairment between noise conditions, we generated another 10,000 permuted datasets for each noise comparison by shuffling the noise labels across trials of the respective conditions. In other words, for comparing LSF and MSF noise, we shuffled the noise labels of trials only with LSF or MSF noise. Then, as previously, we calculated the group level performance of each permuted dataset separately for the two noise conditions in question. We took the minimum value for each permuted dataset in each noise condition. The middle 95th percentile of the differences between the two noise conditions for the permuted datasets served as the critical value. Any observed difference more extreme than the critical value was deemed significant at α = 0.05 for a two-tailed test.

For analyzing the behavioral oscillations, i.e. the periodicity of changes in peripheral discrimination performance, we analyzed the Fourier power of the TPOs tapered by Hanning window. As Contemori et al.^25^, we obtained amplitudes for frequencies ranging from 0 to 20 Hz. We used the previously generated permuted datasets for statistical testing. The permuted datasets followed the same steps as the empirical data. The top 95th percentile power for each condition from the permuted datasets served as a critical value for a one-tailed significance test at α = 0.05. Amplitudes were compared across noise conditions were calculated similarly to the noise comparison analysis by Fourier transforming the permuted datasets created for the noise comparison. The difference between the maximum amplitude in the permuted datasets was used to determine the critical value for testing the real difference for the respective noise conditions. Any observed difference more extreme than the critical value was deemed significant at α = 0.05 for a two-tailed test.

#### Experiment 2

In Experiment 2, we fitted psychometric functions for each observer, eccentricity, background and character condition using the psignifit toolbox for MATLAB^53^ (see Supplementary Fig. S3 for example fitted functions). We defined the contrast threshold of each participant as the contrast yielding 70.7% accuracy according to their psychometric function. Observers with psychometric functions that did not reach the target accuracy were excluded from the analysis of the respective character condition. One additional observer was excluded due to poor fixation throughout the experiment. After exclusions, seven observers had valid thresholds only in one of the two character conditions. Because of this we conducted a linear mixed model (LMM) with observer as random effects and eccentricity (0° vs. 10°), noise frequency (LSF vs. MSF), and character (Latin vs. Korean) as fixed effects. In addition to the main effects and interaction reported in *Results*, the model yielded no significant interaction between character and eccentricity (*t*(119) = 0.346, *p* = 0.73), or between character and noise frequency (*t*(119) =  − 0.267, *p* = 0.79) or between character, eccentricity, and noise frequency (*t*(119) = 0.326, *p* = 0.745).

### Supplementary Information

Supplementary Information.

## Data Availability

Data generated during this study is available at 10.5281/zenodo.10657044. None of the experiments was preregistered. Correspondence and material requests should be addressed to N.G.
