# Supplementary material for: Frequency-specific and periodic masking of peripheral characters by delayed foveal input
Source: Sci Rep. 2024 Feb 26;14:4642. doi: 10.1038/s41598-024-51710-7 (PMC10897220; doi:10.1038/s41598-024-51710-7)
Supplement: Supplementary file 1 — Supplementary Information. [file 41598_2024_51710_MOESM1_ESM.docx]

# Supplementary materials

## *Experiment 1: Comparison to baseline no-noise condition*


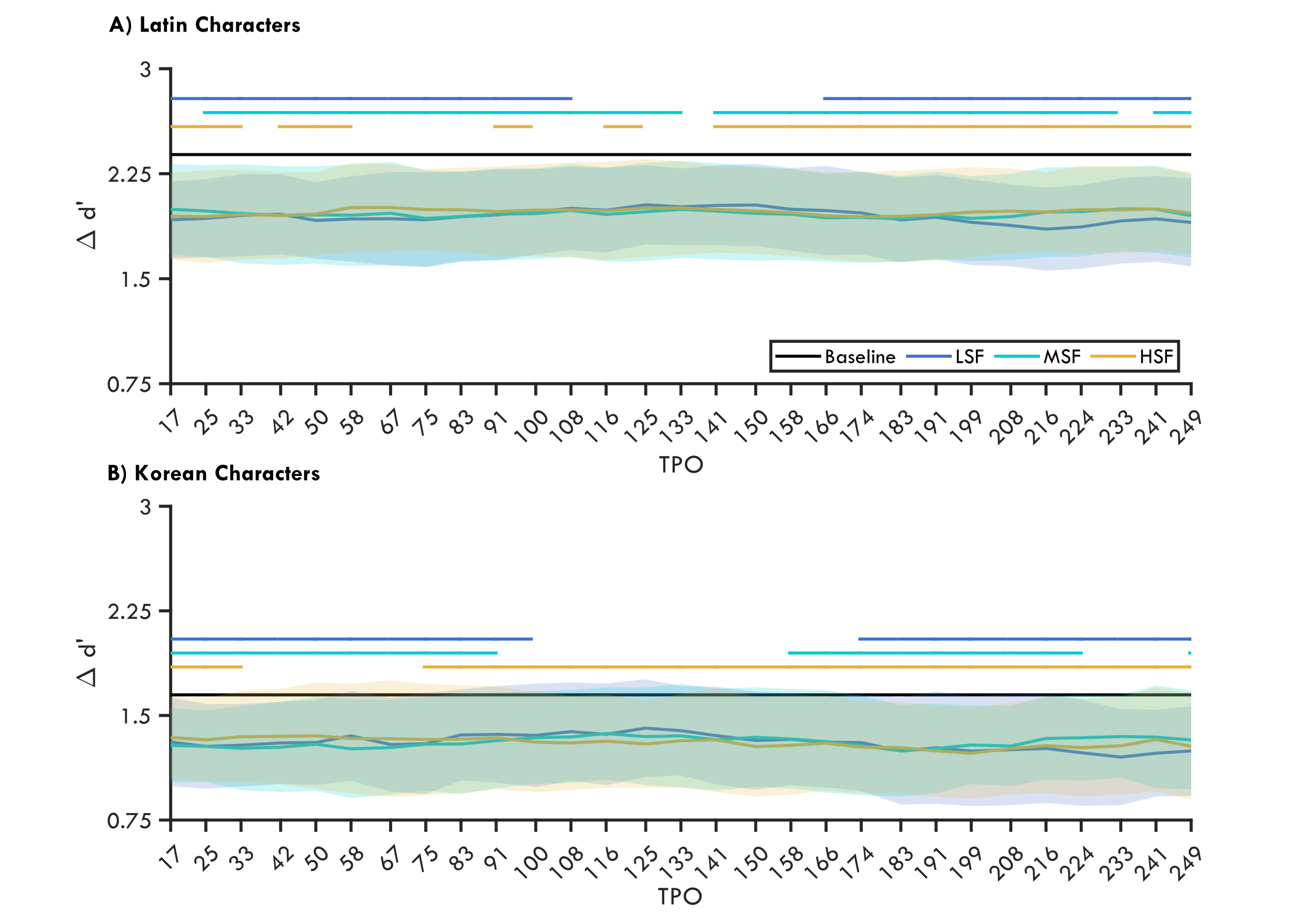


Supplementary Figure 1. Character discrimination performance for Latin (A) and Korean (B) characters with foveal LSF, MSF, and HSF noise relative to the no-noise performance. The black horizontal lines show the no-noise baseline performance. Shaded areas show 99,95% confidence interval corresponding to the alpha level used for statistical analyses (α= 0.0007, for 3 × 25 comparisons in each character condition). The upper horizontal bars indicate SOAs with significant reductions in performance compared to baseline.

Previous studies have used two methods for identifying the time window of foveal-feedback^13,26^. In the results section, we used the method of comparing the different SOAs with each other. The other method entails comparing SOAs to a no-noise baseline condition (e.g., Fan et al.^9^). Here, we re-analyzed the data from Experiment 1 using the other method and provide a short discussion.

We compared each SOA for each noise and character condition to observers’ no noise baseline performance (Supplementary Fig 1). Bonferroni corrected paired-sample t-tests revealed multiple effective SOAs that vary across noise and character conditions. Both conditions LSF noise produced the strongest reduction for similar SOAs. For both characters, we observed a performance reduction around an SOA of 30 within a window of 10-60 ms and around an SOA of 240 within a window of 10-70 ms. The timeframe of the first episode of foveal-feedback is consistent with Weldon et al.^13,26^ while the second episode is consistent with Fan et al.^9^ and Ramezani et al.^27^. Therefore, our results capture the wide range of reports from the previous studies. Whereas MSF noise were effective within smaller time windows around 80 and 170 SOAs around 10-30 ms. Lastly, HSF noise produced inconsistent episodes of performance reductions. However, an episode of reduction starting around an SOA of 170 was common for the two characters. Taken together, in contrast to the SOA comparison method in Results section, baseline comparison suggests inconsistent time windows of foveal-feedback across different noise and character conditions. However, the difference between the two methods is not surprising. When the noise conditions are compared to the no-noise condition, there is an inherit assumption that any change in performance is due to foveal-feedback, which may not be necessarily the case. In fact, using this method Fan et al.^9^ found a reduction of performance at 50 ms SOA as we did. They attributed this reduction to attention as the noise presentation coincided with the peripheral target presentation. This is a more plausible explanation as TMS results show that foveal-feedback takes place around 300 ms after target onset^8^. While this method is prone to false positives, comparing SOAs with each other can be over conservative as it would yield a null result if there are multiple episodes of foveal-feedback causing similar amounts of reduction. Therefore, our analysis in the main results is more likely to show the time window of foveal-feedback when it is strongest.

## *Experiment 1: Control Measures*


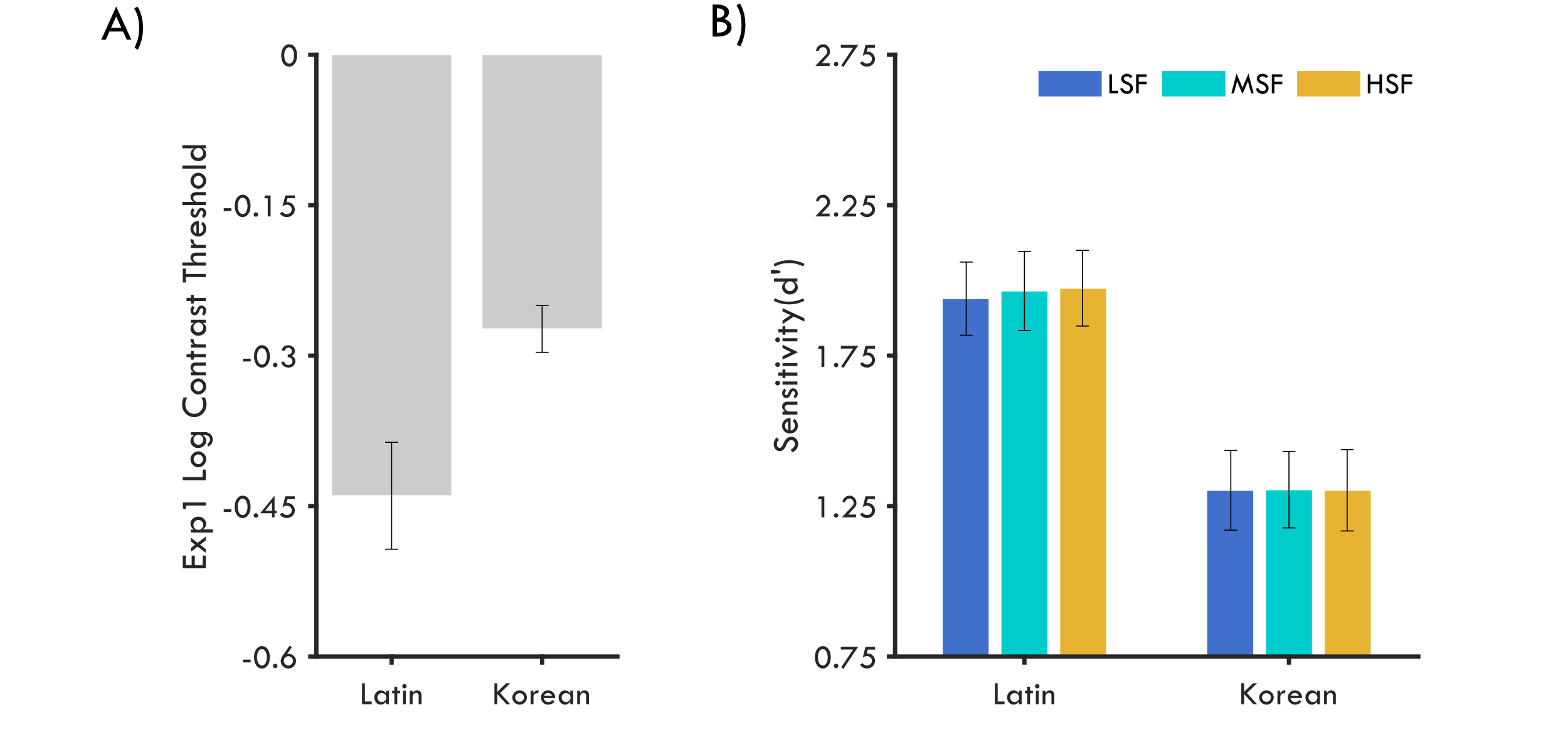


Supplementary Figure 2. Mean Log character contrast thresholds (A) and sensitivity across foveal noises (B). Error bars show 95% confidence intervals.

Observers in both Korean and Latin character conditions performed the initial thresholding procedure. Korean characters required more contrast to reach the same target threshold as Latin characters (Supplementary Figure 2, *t*(55)= 4.551, *p* < 0.001). We conducted a 2 (Character: Korean, Latin) × 3 (Noise: Low, Medium, High) mixed design ANOVA to compare the discrimination performance across conditions. As expected, the discrimination performances did not significantly differ between noise conditions (*F*(2,110)= 0.516, *p* = 0.598) and there was no interaction between noise and character conditions (*F*(2,110)= 0.48, *p*= 0.620). However, discrimination performance was significantly higher for Latin than Korean characters (*F*(1,55)= 38.651, *p* < 0.001) despite the higher contrast for the Korean characters.

# *Experiment 2: Character Contrast Thresholds*


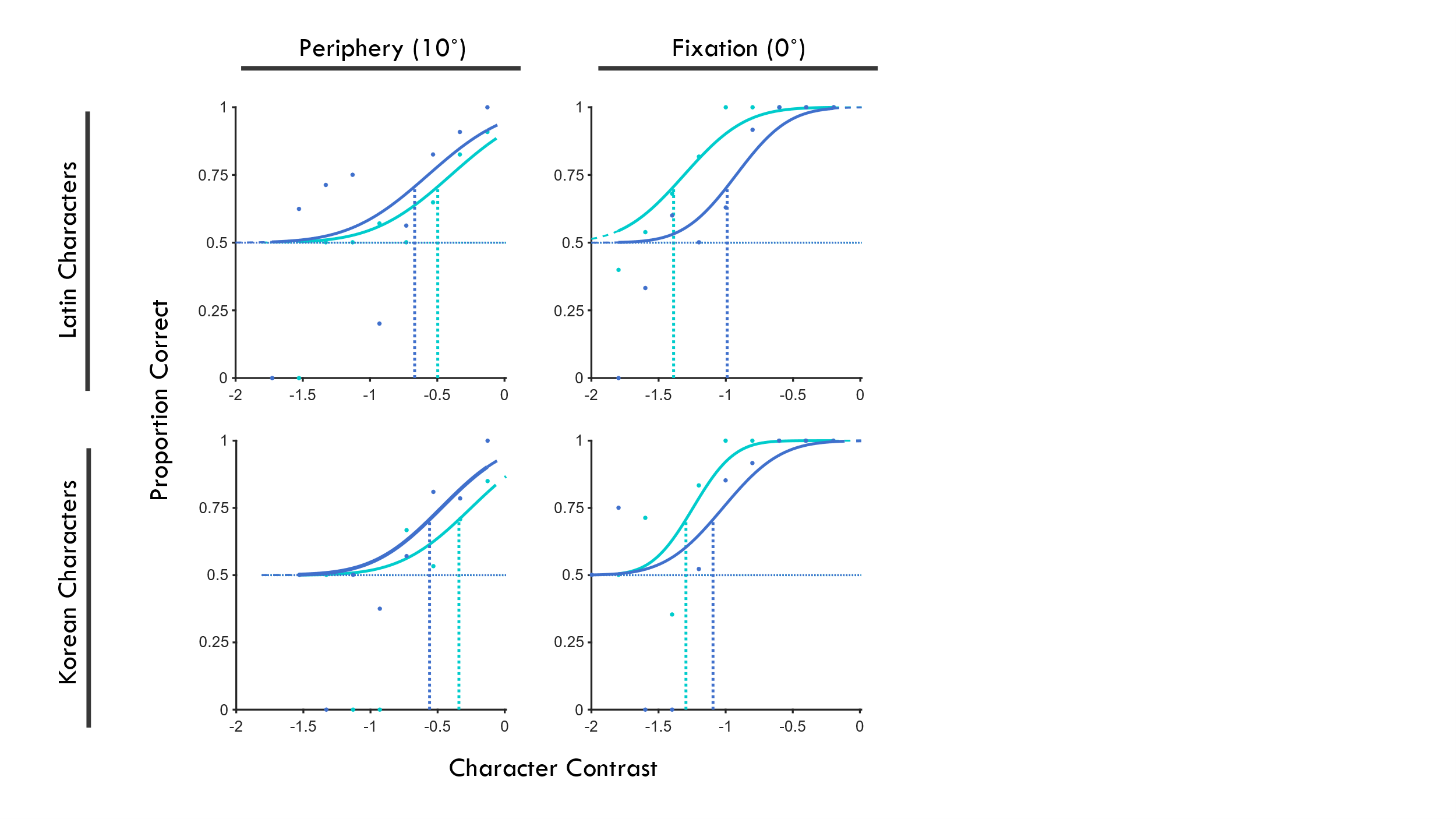


*Supplementary Figure 3*. Example psychometric functions for estimating the contrast of Korean and Latin characters presented in the periphery and near the fovea. Characters embedded in LSF and MSF noise are represented with light and dark blue, respectively. The horizontal dashed lines show the chance level performance. The vertical dashed lines show 70.7 % discrimination performance.

In Experiment 2, observers were asked to discriminate Latin and Korean characters that were embedded either in LSF or MSF noise in the periphery or near the fovea. Character contrast in each trial was varied according to a 2-up-1-down staircase procedure with two staircases starting from the opposite ends of the contrast range. We estimated the contrast threshold of characters presented in both locations by fitting a psychometric function for each observer and condition (see Supplementary Figure 3).
